# Supplementary material for: Grafting of Cyclodextrin to Theranostic Nanoparticles Improves Blood-Brain Barrier Model Crossing
Source: Biomolecules. 2023 Mar 22;13(3):573. doi: 10.3390/biom13030573 (PMC10046162; doi:10.3390/biom13030573)
Supplement: Supplementary file 1 [file biomolecules-13-00573-s001.zip › biomolecules-2235322-supplementary.pdf]

Grafting of Cyclodextrin to Theranostic Nanoparticles Improve Blood-Brain Barrier Model Crossing

Antonino Puglisi [1,*], Noemi Bognanni [1,2], Graziella Vecchio [2], Ece Bayir [3], Peter van Oostrum [1], Dawn Shepherd [4], Frances Platt [4] and Erik Reimhult [1]

[1] Department of Bionanosciences, Institute of Biologically Inspired Materials, University of Natural Resources and Life Sciences (BOKU), 1190 Vienna, Austria

[2] Dipartimento di Scienze Chimiche, Università degli Studi di Catania, 95125 Catania, Italy

[3] Central Research Testing and Analysis Laboratory Research and Application Center, Ege University Bornova, Izmir 35100, Turkey

[4] Department of Pharmacology, University of Oxford, Oxford OX1 3QT, UK

[*] Correspondence: antonino.puglisi@boku.ac.at

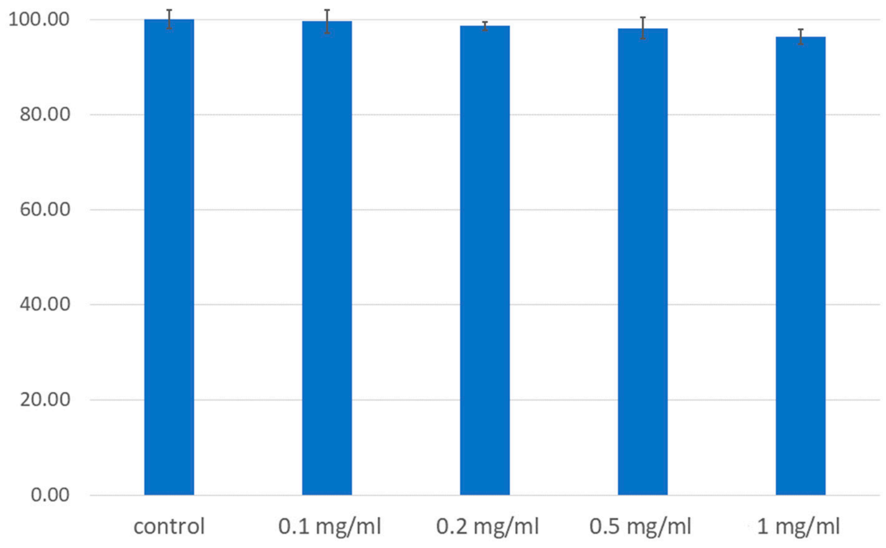

Figure S1-*Resazurin viability assay on Npc1-deficient CHO cells after 24 h incubation with different high concentrations of CySPION and 48 h regeneration compared to a control, showing no significant cytotoxicity.*

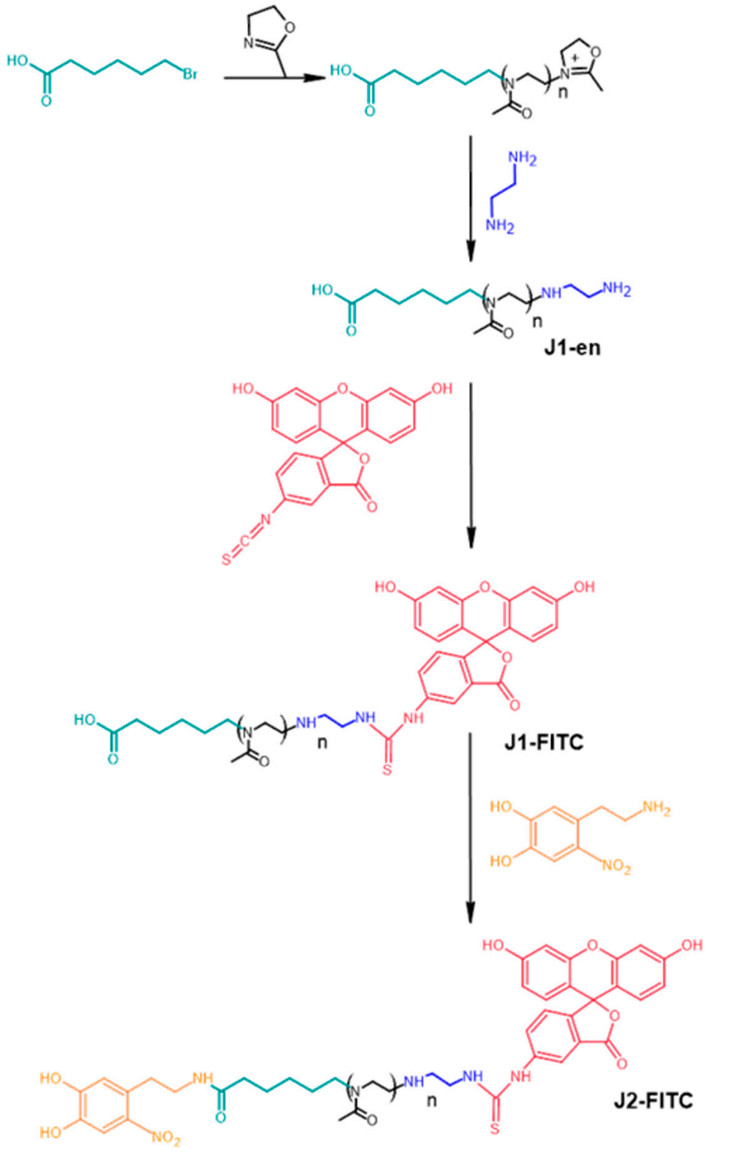

Figure S2-*General scheme of synthesis of the fluorescein-labelled polymer*

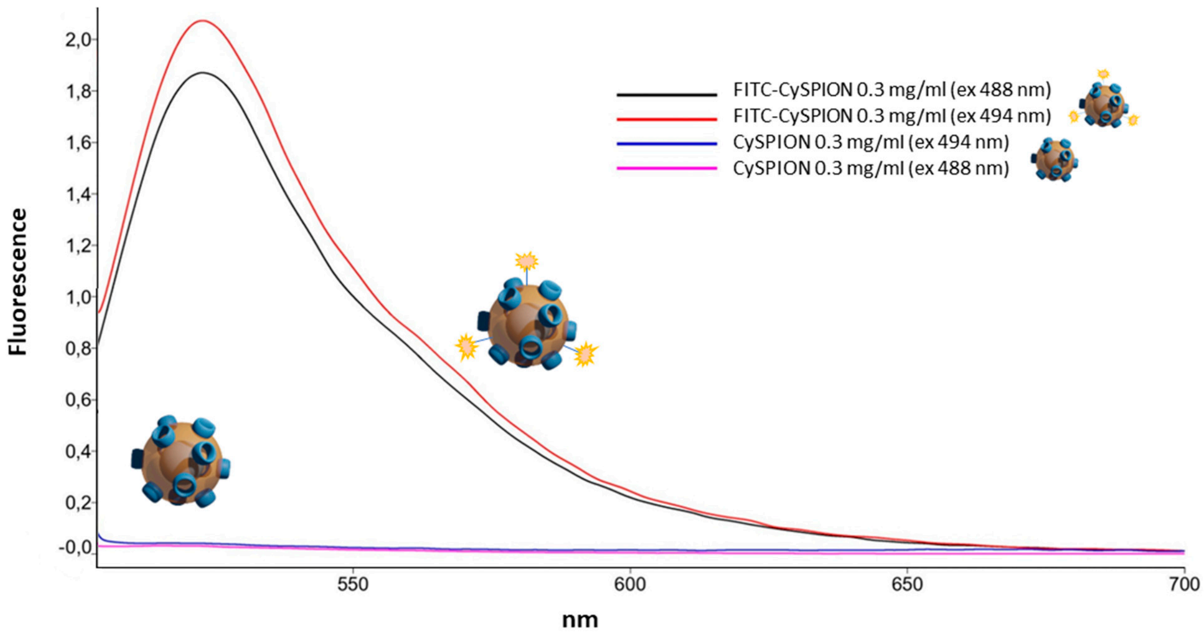

Figure S3 *Fluorescence of CySPION and FITC- CySPION at 488 nm and 494 nm*

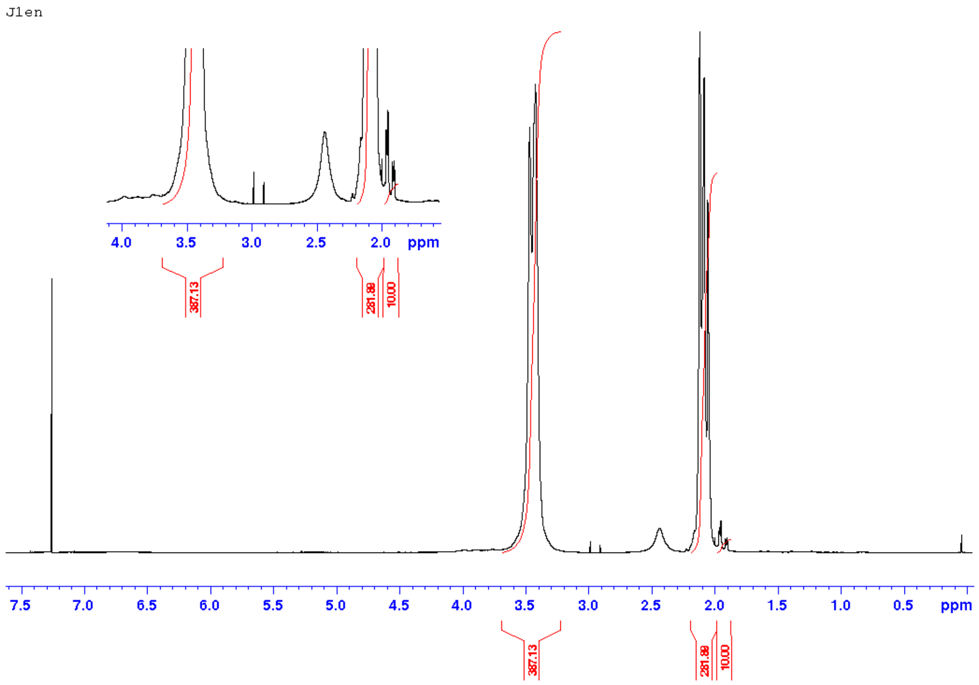

Figure S4 - *^{1}HNMR (300 MHz) of J1-en in $CDCl_3$*

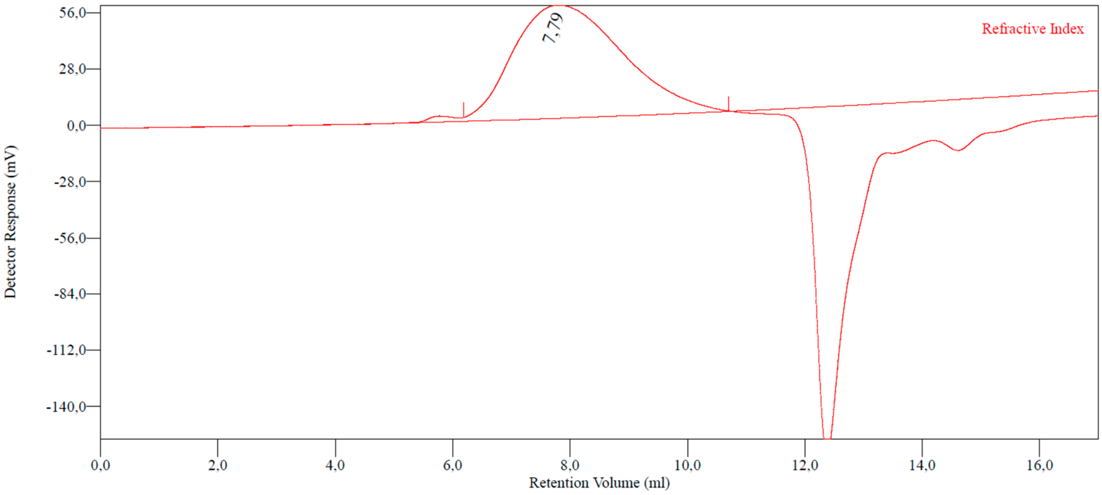

Figure S5 - *GPC of J1-en*

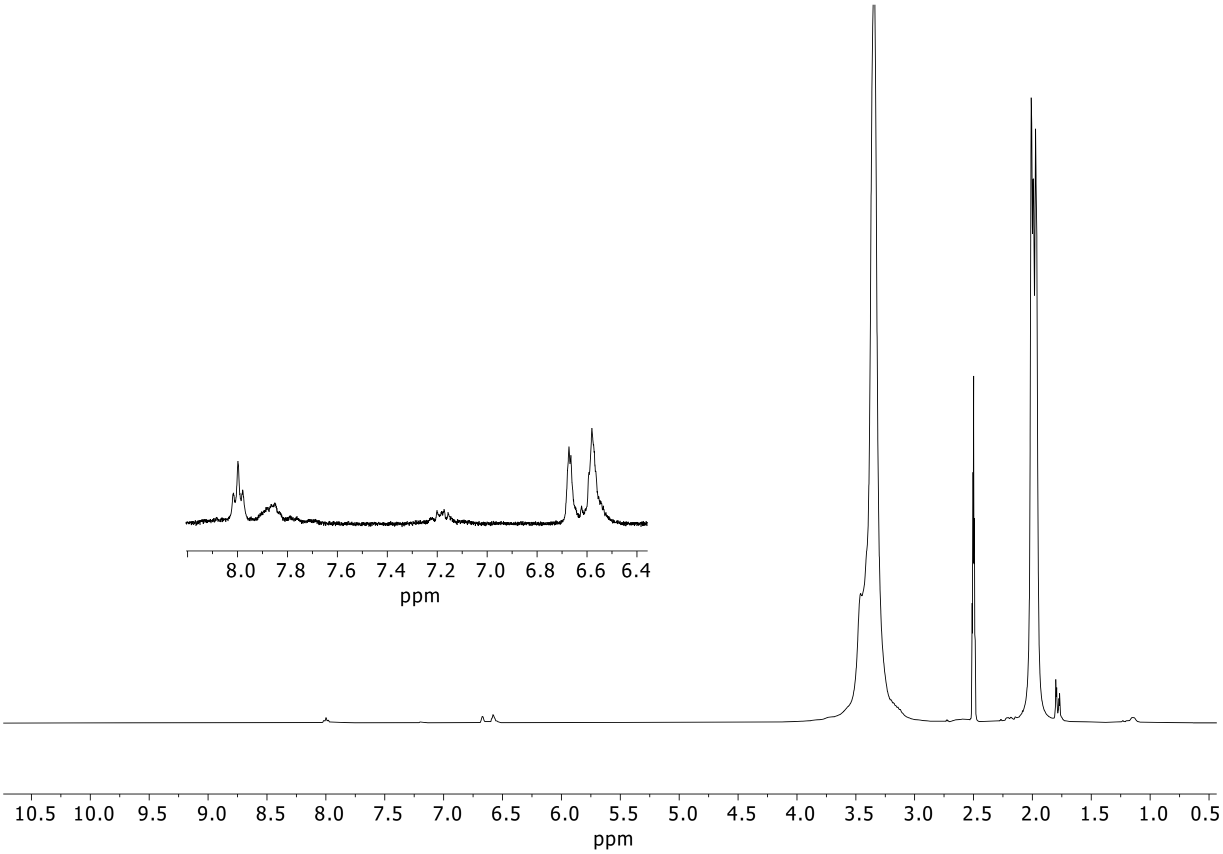

Figure S6 - *^{1}HNMR (300 MHz) of J1-FITC in DMSO*

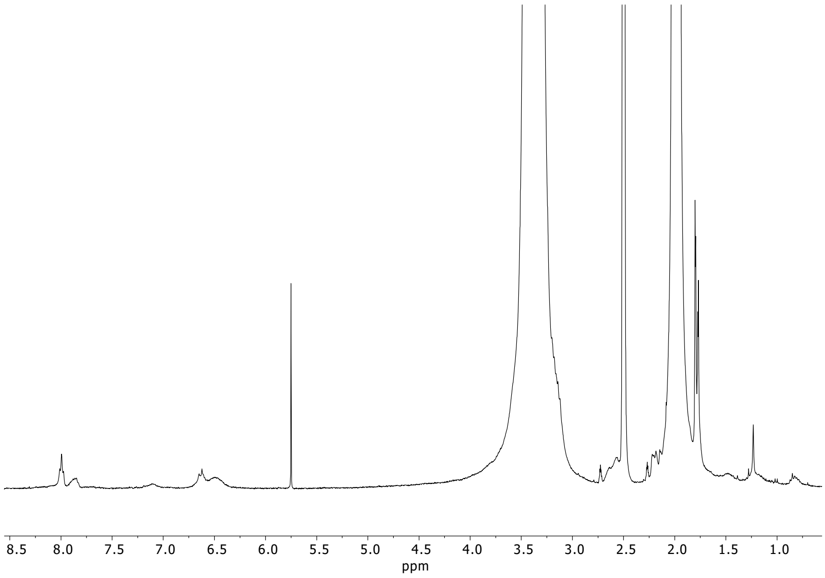

Figure S7 - *^{1}HNMR (300 MHz) of FITC-PMOXA in DMSO*

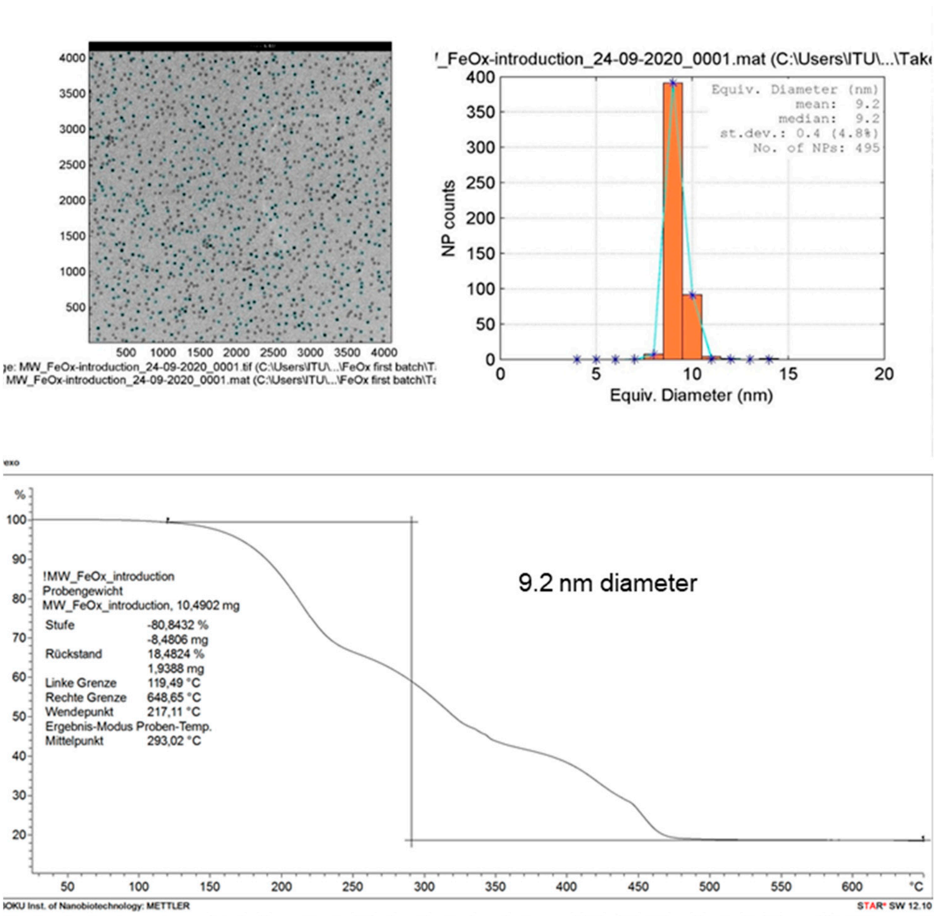

Figure S8 - *TEM and TGA of oleic acid-coated SPIONs obtained via heat-up method*

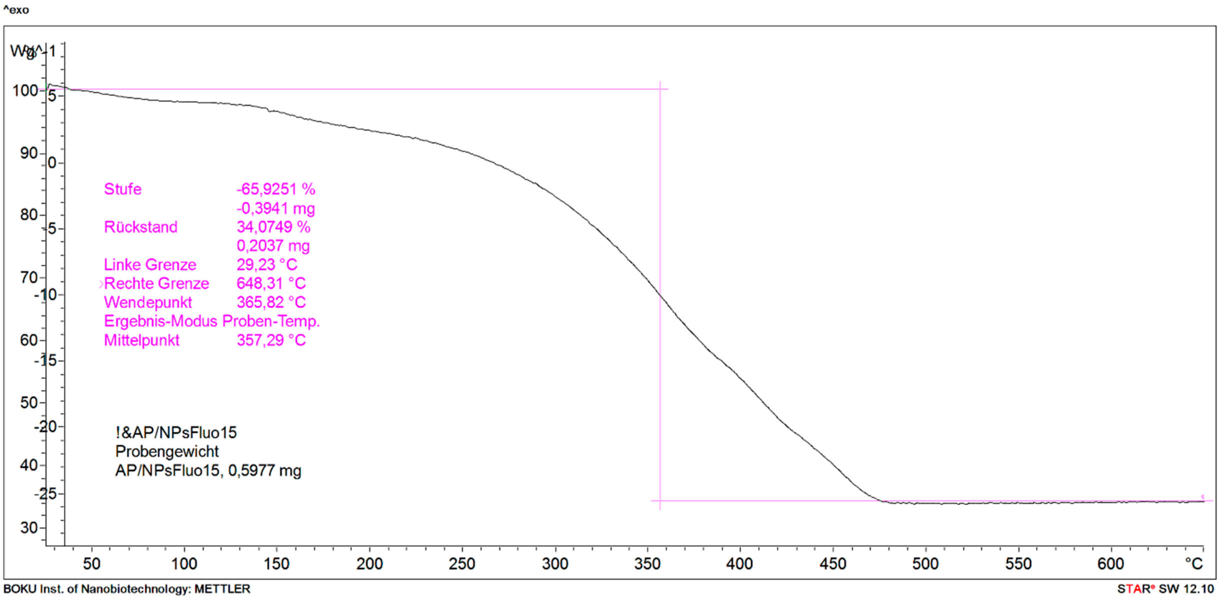

Figure S9 - *TGA CySPIONs dopped with 15% fluorescein-labelled polymer*

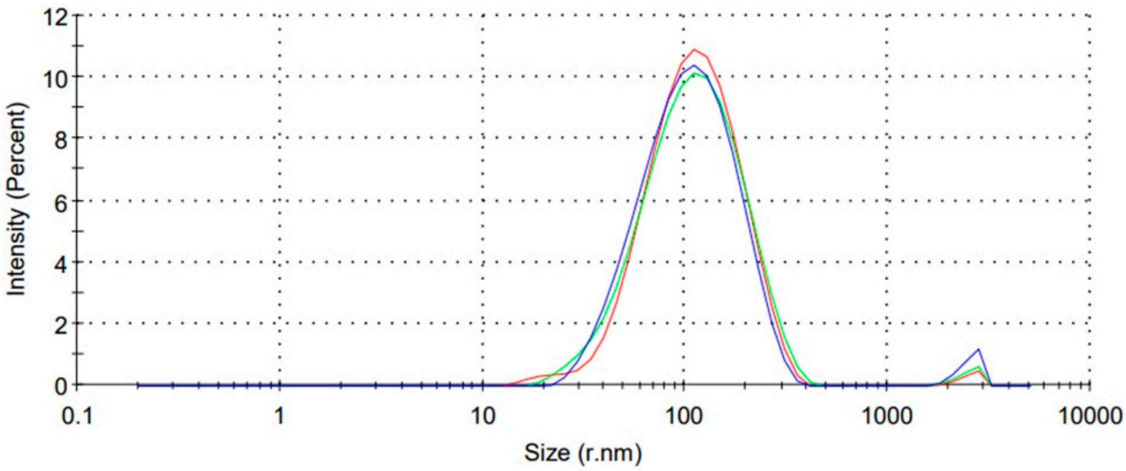

Figure S10 - *Size distribution by intensity for FITC-CySPION. Radius is 120 nm*

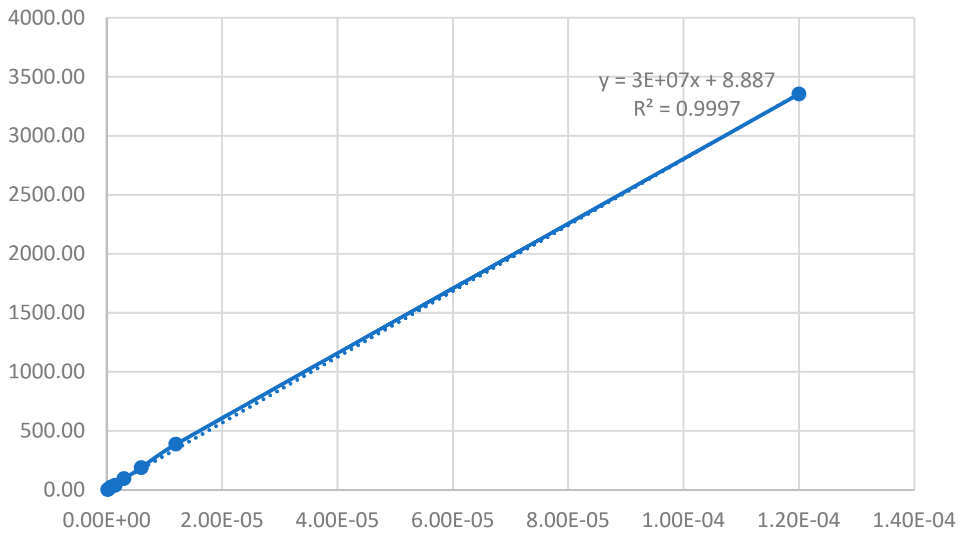

Figure S11 - *Calibration curve of LY*

Table S1 - *Calibration curve of LY at 530nm*

	**Concentration (M)**	**Fluorescence (530nm)**
1	1.20E-04	3353.10
2	1.20E-05	385.40
3	6.00E-06	186.40
4	3.00E-06	93.00
5	1.50E-06	37.10
6	7.50E-07	22.20
7	3.75E-07	7.00
8	1.88E-07	0.00

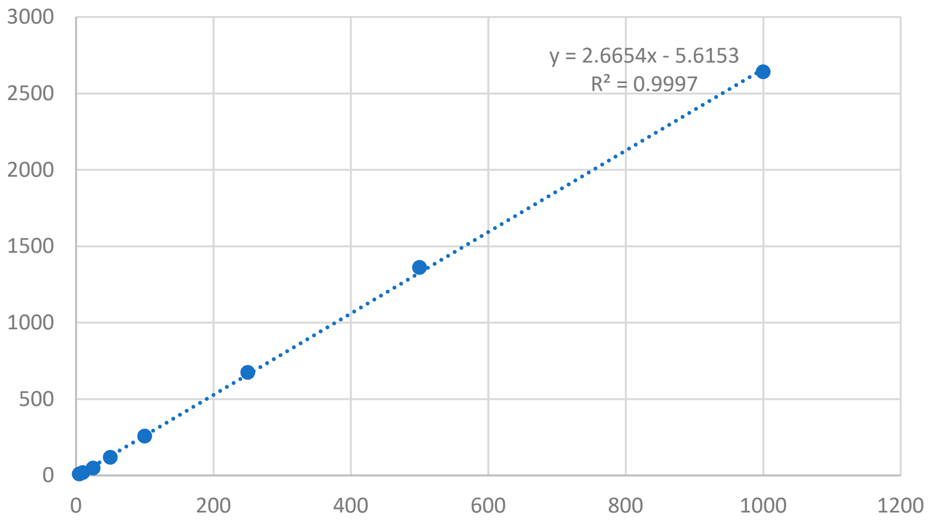

Figure S12 - *Calibration curve of FITC-PMOXA*

Table S2 - *Calibration curve of FITC-PMOXA at 520nm*

	**Concentration FITC-PMOXA (µg/mL)**	**RFU (520 nm)**
1	1000	2640.9
2	500	1361
3	250	673.6
4	100	256.4
5	50	118.3
6	25	48.3
7	10	18.4
8	5	9.1

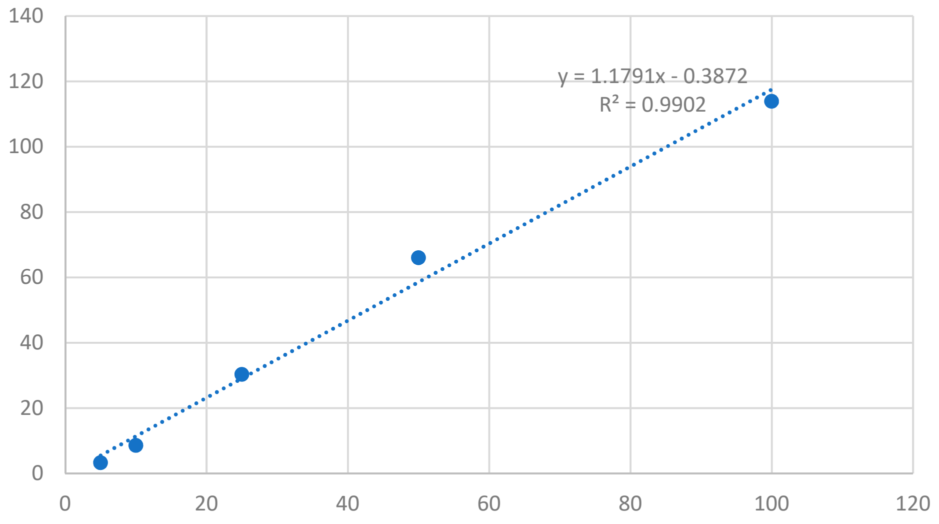

Figure S13 - *Calibration curve of FITC-CySPION*

Table S3 - *FITC-CySPION at 520nm*

	**Concentration FITC-CySPION (µg/mL)**	**RFU (520 nm)**
1	100	113.9
2	50	66
3	25	30.3
4	10	8.6
5	5	3.3

Table S4 - *Permeability values for LY, FITC-PMOXA and FITC-CySPION*

Lucifer Yellow

Time (min)	Permeability (cm/s)
30	1.38536E-06
60	1.65806E-06
90	2.048E-06

FITC-PMOXA

Time (min)	Permeability (cm/s)	Standard deviation
30	1.25396E-06	6.88833E-07
60	2.4988E-06	4.56429E-07
90	1.66836E-06	3.23723E-07

FITC-CySPION

Time (min)	Permeability (cm/s)	Standard deviation
30	3.77893E-06	8.27035E-07
60	3.06986E-06	7.23057E-07
90	2.27464E-06	2.62875E-07
